# Evaluation of antifungal and cytotoxic activity of *trans*-Chalcone and α-Solanine

**DOI:** 10.1186/1753-6561-8-S4-P36

**Published:** 2014-10-01

**Authors:** Tatiana Takahasi Komoto, Gabriel Silva, Tamires Bitencourt, Bruna Azevedo Cestari, Mozart Marins, Ana Lúcia Fachin

**Affiliations:** 1 Universidade de Ribeirão Preto, Ribeirania, Brazil

## Background

Dermatophytes are adapted to grow in keratinized tissues such as skin, nail and hair. *Trichophyton rubrum *is the most frequent cause of dermatophytosis in Brazil and in the world [1]. Despite its incidence there are only a limited number of antifungal drugs available for clinical use and some drugs are highly toxic to humans. In this regard, chalcones and alkaloids are phytochemical products which provide a rich source of chemical diversity for the development of new antifungals. Chalcones inhibit the biosynthesis of the cell wall and activity of fatty acid synthase in yeast [2,3]. The glycoalkaloid α-Solanine purified from potate sprout presents antifungal activity by altering cell membrane integrity and inhibition of sporulation [4]. The aim of the present study was to evaluate the minimum inhibitory concentration (MIC) and cytotoxicity (by MTT) of *trans*-Chalcone and α-Solanine toward strain MYA3108 of *T.rubrum *and the keratinocyte cell line HaCat, in order to evaluate the potential use of these phytochemicals against fungal skin infection.

## Materials and methods

The antifungal activity of the compounds was determinated by using the M38-A microdilution technique according to the Clinical and Laboratory Standards Institute [5] toward strain *T.rubrum *for 7 days at 28°C. Keratinocytes were cultures in RPPMI supplemented with 10% fetal calf serum and incubated at 37°C and 5% CO_2_. Cells were plated (2.5x10^5 ^cells/mL) in a 96-well tray 24 h prior to the beginning of the experiment. After addition of several concentrations of natural compounds or the vehicle, cells were analyzed after a period of 24 h using the MTT assay.

## Results

The MICs of α-Solanine and *trans*-Chalcone were 7.8 µg/mL, showing effectiveness against *T. rubrum*, while the inhibition of the HaCat cell line by *trans*-Chalcone (7.8 µg/ml) and α-Solanine (50 µg/ml) were 45.78% and 68.86% respectively.

## Conclusions

Finally, the α-Solanine is a potential candidate for the development of new antifungal drugs against *T. rubrum*, due to its significant antifungal activity and lower citotoxicity for human keratinocytes.

## Financial support

This work was supported for CAPES and FAPESP.
